# Retraction of “miR-223-3p alleviates TGF-β-induced epithelial-mesenchymal transition and extracellular matrix deposition by targeting SP3 in endometrial epithelial cells”

**DOI:** 10.1515/med-2023-0889

**Published:** 2023-12-31

**Authors:** Yanling Chen, Dongyan Sun, Di Shang, Zhihe Jiang, Pan Miao, Jian Gao

**Affiliations:** Department of Obstetrics and Gynecology, School of Medicine, Wuhan University of Science and Technology, Wuhan 430072, Hubei, China; Department of Gynecology, Maternity and Child Health Care Hospital of Hubei Province, 745 Wuluo Road, Wuchang District, Wuhan 430000, Hubei, China; Yangtze University Health Science Center, Jingzhou 430199, Hubei, China

Retraction of: Chen Y, Sun D, Shang D, Jiang Z, Miao P, Gao J. miR-223-3p alleviates TGF-β-induced epithelial-mesenchymal transition and extracellular matrix deposition by targeting SP3 in endometrial epithelial cells. Open Medicine 2022;17(1):518–526, DOI: 10.1515/med-2022-0424.

This article has been retracted by the publisher due to irregularities in the figures and lack of clarity regarding the origin of the images. As a result, the editors cannot guarantee the legitimacy of the presented data.

